# Advancing Mental Healthcare Access for Older People: Results from the National Medicare ACCESS Initiative

**DOI:** 10.1093/geroni/igaf122.3044

**Published:** 2025-12-31

**Authors:** Jordan Westcott, Alexis Isaac, Matthew Fullen

**Affiliations:** University of Tennessee at Knoxville, Knoxville, Tennessee, United States; Virginia Tech, Blacksburg, Virginia, United States; Virginia Tech, Blacksburg, Virginia, United States

## Abstract

Historically, older adults have had limited access to mental health services despite having unmet mental health needs (Adepoju et al., 2018). After more than 30 years of advocacy, licensed mental health counselors and marriage and family therapists became eligible for participation in the Medicare program on January 1, 2024 (Consolidated Appropriations Act, 2023), leading more than 60,000 new mental health professionals to enroll in the Medicare program (CMS.gov, n.d.). Therefore, the authors developed the national Medicare ACCESS research initiative to understand initial experiences, motivating factors and concerns, and gerontological counseling competencies with the Medicare program among the newly enrolled mental health provider workforce. Among 127 respondents, 87.4% had provided services to one or more older adult clients since enrolling in the Medicare program. Most highly rated motivating factors for Medicare enrollment included expanding mental healthcare access for older adults (60.8% rated as high importance) and supporting people in one’s community (57.6%), and providers were most concerned about Medicare reimbursement rates (68.0%) and compliance with Medicare policies (56.0%) after enrolling. These findings will be contextualized with textual responses illuminating provider perspectives. Additionally, provider age (b = .186, p = .035) and provider attitude toward aging (b = -.319, p < .001) had significant linear relationships with gerontological counseling competencies, R2 = .164, F(2, 114) = 11.16, p < .001 with a moderate effect size (f2 = .196; Cohen, 1988). Implications for provider enrollment efforts, service delivery for older adults seeking mental healthcare, and Medicare advocacy will be discussed.

